# Scientific and targeted prevention and control measures to optimize COVID‐19 response

**DOI:** 10.1002/hcs2.33

**Published:** 2023-01-12

**Authors:** Released by the Taskforce for Joint Prevention and Control Mechanism for COVID‐19 under the State Council and the National Health Commission of P.R. China on November 11, 2022

**Keywords:** China, COVID‐19, disease prevention and control, public health policy, the 20‐point measures

## Abstract

Since the outbreak of the COVID‐19 pandemic, under the strong leadership of the Central Committee of the Communist Party of China and President Xi Jinping, China has made firm decisions to prioritize people's lives and health. Efforts have been made to avoid foreign imported infection and domestic rebound of COVID‐19 cases; the “dynamic zero COVID” policy has been executed without wavering; and the prevention and control measures have been optimized and improved in response to changing circumstances, actively responding to the impact caused by multiple waves of COVID‐19 surge globally. China has made extraordinary efforts to safeguard people's lives and health. Meanwhile, prevention and control measures were timely updated to coordinate with economic and social development. On November 10, the Standing Committee of the Central Political Bureau held a meeting to discuss the latest situation of the COVID‐19 pandemic in China. Twenty measures (referred to as “**the 20‐point measures**“) were officially announced to further optimize the COVID‐19 response. The latest prevention and control measures involve the requirements and guidance for isolation at home, high‐risk area delineation, nucleic acid testing, international inbound flights and travelers, vaccination rollout, medical resource preparedness, and protection for special places and vulnerable population.

**Abbreviations**
COVID‐19: coronavirus disease 2019
Ct: cycle threshold

## 1 RAISE THE POLITICAL AWARENESS FOR SCIENTIFIC AND TARGETED PREVENTION AND CONTROL OF THE PANDEMIC

The global pandemic caused by the SARS‐Cov‐2 virus remains prevalent because of evolving variants of the virus. Recently, an increasing number of outbreaks have taken place in China. China has a vast population, a large number of vulnerable individuals, unequal regional development, and relatively limited medical resources. In addition to the mutating variants, winter weather and other factors are contributing to the current COVID‐19 outbreaks that have recently affected various areas. Given the severity and complexity of the issue, China must retain determination and implement scientific pandemic prevention and control strategies. Administrative authorities at all levels should consistently and accurately execute the “dynamic zero COVID” policy to avoid foreign imported infection and domestic rebound of COVID‐19 cases. The goal is to achieve a balance between managing the pandemic and stabilizing the economy. These modifications do not imply loosening prevention and control, let alone liberalizing or “lying flat” (giving up the battle against the pandemic and doing nothing to respond), but rather adjusting to the new challenges posed by the ever‐changing variants of the SARS‐Cov‐2 virus. Scientific and precision prevention and control measures will safeguard people's lives and health while also limiting the impact of the pandemic on social and economic development.

## 2 TO IMPLEMENT THE POLICY ENACTED BY THE PARTY CENTRAL COMMITTEE, THE FOLLOWING MODIFICATIONS SHALL BE MADE TO THE NATIONAL COVID‐19 PREVENTION AND CONTROL MEASURES

(1) For close contacts of confirmed Covid‐19 cases, the requirement of “7‐day centralized quarantine + 3‐day health monitoring at home” will be reduced to “5‐day centralized quarantine + 3‐day home isolation.” Close contacts should be assigned a health status code and must not leave their home during the isolation period. Nucleic acid testing should be performed on Days 1, 2, 3, and 5 of the centralized quarantine period, as well as Days 1 and 3 of the home isolation period.
(2) Confirmed cases' close contacts should be identified in a timely and accurate fashion. Close contacts of close contacts will no longer be traced.
(3) For individuals that have traveled from high‐risk areas, the requirement of “7‐day centralized quarantine” will be reduced to “7‐day home isolation.” During isolation, people from high‐risk areas should be assigned a health status code and must not leave their home. Nucleic acid testing should be performed on Days 1, 3, 5, and 7 of the home isolation period.
(4) The regional classifications will be adjusted from “high, medium, and low risk” to “high and low risk” to minimize the number of restricted persons. In principle, the places in which confirmed cases live, work, and visit frequently are designated as high‐risk areas. High‐risk areas are generally delineated by units and buildings, the scope of which may not be expanded at will. Areas in the county, city, or district other than the high‐risk areas are delineated as low‐risk areas. If no new infections are reported in a high‐risk area for 5 consecutive days, it should be downgraded to a low‐risk area. High‐risk areas that meet the requirements for lifting risk control should be lifted in a timely manner.
(5) For individuals who have finished working in high‐risk positions, the requirement of “7‐day centralized quarantine or 7‐day home isolation” is reduced to “5‐day health monitoring at home,” and isolated personnel will be assigned a health status code. Nucleic acid testing should be performed on Days 1, 3, and 5 of home isolation. Leaving the monitoring location should be avoided as much as possible; in cases in which leaving is necessary, individuals should avoid crowded public places and public transit.
(6) Areas without outbreaks should perform nucleic acid testing for high‐risk occupations and high‐risk personnel in accordance with the scope defined in the COVID‐19 pandemic prevention and control protocol (Version 9) [1], which should not be expanded at will. In general, mass nucleic acid testing should no longer be organized at the level of administrative areas, but only when the origin of infection and the chain of transmission are unknown, or if the community has a prolonged transmission time, and so on. The requirement for nucleic acid testing should be further regulated to avoid multiple testing.
(7) The “circuit breaker” mechanism for inbound flights will be lifted, and the requirement of two negative nucleic acid test results within 48 h before boarding will be reduced to one negative nucleic acid test result within 48 h before boarding.
(8) International inbound business people and sports teams should be transferred point‐to‐point to the isolation‐free closed‐loop management area (“closed‐loop bubble”) for business, training, and competition activities. During this time, the individuals involved will be assigned health status codes and will be restricted from leaving the management area. Personnel from China are required to receive a booster vaccine before entering the management area, and are subject to isolation or health monitoring afterward on the basis of the assessed level of risk.
(9) International inbound personnel with nucleic acid test results showing a cycle threshold (*Ct*) value < 35 will be confirmed as COVID‐positive. Individuals with nucleic acid test results with a Ct value of 35–40 will be subjected to risk assessment at the end of quarantine; if considered previously infected, two nucleic acid tests within 3 days of home quarantine are required.
(10) For international inbound personnel, the requirement of “7‐day centralized quarantine + 3‐day home health monitoring” will be reduced to “5‐day period of centralized quarantine + 3‐day period of home isolation,” during which the individuals should be assigned a health status code and must not leave their home. After completing quarantine at the first point of entry, no quarantine shall be enforced at other destinations. Nucleic acid testing should be performed on Days 1, 2, 3, and 5 of the centralized quarantine period, as well as Days 1 and 3 of the home isolation period.
(11) Strengthen the preparedness of medical resources. A graded treatment plan should be developed to clarify admission criteria for infected persons of different clinical severity. Plans for medical institutions in response to hospital outbreak or infection among healthcare professionals should also be outlined. Professional training of medical staff should be arranged. Each medical institution should increase the number of beds for inpatient and critical care to enhance the treatment capacity.
(12) Promote vaccination and improve vaccine booster coverage, especially for the elderly population. Accelerate the development of monovalent or multivalent vaccines with broad‐spectrum protection, as well as their regulatory approval.
(13) Stockpile COVID‐19 treatment‐related medications. Ensure good supply reserves for those at risk of serious morbidity and the older people. Stock up on effective Chinese medicine prescriptions. Prepare an adequate supply of emergency medications and medical equipment.
(14) Enhance the protection of critical institutions and vulnerable populations. Collect data on the characteristics of older people, patients with underlying conditions, pregnant women, and hemodialysis patients, then implement health protection programs accordingly. Pay specific attention to the management of facilities that house vulnerable populations, such as nursing homes, psychiatric hospitals, and orphanages.
(15) To limit the spread of the pandemic and speed up the emergency response, surveillance and early warning systems should be built through multiple channels and a multipoint trigger mechanism should be further developed. People traveling across provinces should be subjected to a “test upon arrival.” Infected people must be reported in line with the legislation; epidemiological investigations and control of people at risk must be carried out in a timely fashion. Early discovery, reporting, isolation, and treatment can help to prevent the disease from becoming widespread and prolonged.
(16) Relevant departments in localities are required to rectify oversimplified or one‐size‐fits‐all approaches and excessive policy steps taken during pandemic control. Local authorities are responsible for implementing the national prevention and control policy. Unauthorized school closures, business suspensions, traffic blockades, arbitrary or lengthy lockdowns, and clinic closures should be strictly prohibited. Incidences with serious consequences will be identified and prosecuted in accordance with the law. The Health Commission, Centers for Disease Control and Prevention, Ministry of Education, Ministry of Transportation, and other departments shall supervise and guide the relevant industry.
(17) Quarantined personnel should be provided with good services and logistical support. The local authorities should establish working groups and make timely plans to ensure the supply and distribution of essential goods. The basic information and needs of the residents, especially older people living alone, children in distress, pregnant women, and people with basic diseases, should be registered. Courier delivery service in restricted areas should function to guarantee the supply of necessities. Local administrations should guide the community to establish direct channels with medical institutions and pharmacies, and the community should have designated vehicles for transfer and other services. Medical institutions are responsible for the timely treatment of acute and critical cases, and shall not refuse to provide medical treatment for any reason. Mental health services should be provided to quarantined persons, particularly older people, the sick and disabled, and other vulnerable groups.
(18) Refined pandemic prevention and control measures should be applied on campus. Schools and local administrations should work in concert to prioritize quarantine transit, nucleic acid testing, contact tracing investigations, environmental disinfection, and supply of essential goods on campus. All schools should strictly implement the prevention and control measures specified by the national government and education departments; additional control measures without authorization are strictly prohibited. The Ministry of Education and education departments at all levels should organize working groups to investigate outstanding issues such as arbitrary campus closures, excessively long closures, prolonged absence of offline teaching, lack of essential goods, and inconsistent measures applied for teachers, students, and employees. Neither ineffective nor excessive pandemic control measures will be allowed. Education departments at all levels shall establish grievance mechanisms and hotlines for urgent demands of teachers and students, and provide timely responses and feedback.
(19) Prevention and control measures for enterprises and industrial parks. The local authorities should organize working groups to enforce pandemic control measures on the basis of the situation of enterprises (including private enterprises) and industrial parks under their management. Leadership of enterprises and industrial parks, employee groups, and front‐line workers should be aware of their responsibilities in pandemic prevention and control. Employees of enterprises are subject to strict health status checks before returning to work. Special attention should be given to the life, health, and work shifts of the workforce in critical professions and positions. Third‐party outsourced employees and other personnel entering or leaving the industrial park shall be strictly monitored. During the pandemic, efforts should be made to ensure the smooth flow of logistics. The administration should not request unauthorized shutdown of enterprises that are critical to the overall industrial chain and the supply of livelihood essentials. The implementation of white‐listing mechanisms should be ensured.
(20) Individuals stranded by the pandemic should be provided with proper arrangements. Places in which outbreaks occur should undertake timely and precise delineation of risk areas and allow people who are not in high‐risk areas to leave in an orderly way with proper protection. Customized evacuation plans should be designed in places with larger numbers of stranded people. Communication and collaboration between concerned authorities at departure and destination places should be strengthened, and transportation assistance should be provided by the transportation departments, civil aviation, and national railways. Destinations should not refuse to accept the return of stranded people, and should manage the returning people in accordance with the regulations.

## 3 STRENGTHEN RISK PRECAUTION AND PUT MEASURES INTO PRACTICE

All departments should adhere to bottom‐line and problem‐oriented thinking to ensure an orderly handover when implementing the adjusted measures. The following procedures in risk management are highlighted in the new policy.

### 3.1 Management of people at risk

To avoid cross‐infection, local management should manage the centralized quarantine facilities effectively. Individuals who meet the requirement should be able to isolate at home in accordance with the regulations. When a person is diagnosed as COVID‐19‐positive during isolation, the risk of transmission should be assessed, and the related people at risk should be traced to prevent the disease from spreading; those who do not meet the requirement for home isolation should stay in centralized quarantine facilities. Closed‐loop management and personal protection should be strengthened for personnel working in high‐risk positions to prevent them from contracting COVID‐19 at work.

### 3.2 Delineation of high‐risk areas

In the event of an outbreak, local authorities should promptly delineate high‐risk areas and announce this to the public. Lockdowns of high‐risk areas should be targeted to specific buildings or units. In cases where the risk of transmission is unclear or there is widespread community transmission, the delineation of high‐risk areas may be expanded accordingly.

### 3.3 Adjusted policy for inbound travelers

Local authorities should have sufficient resources to manage centralized isolation facilities. During home quarantine, inbound visitors should be closely regulated. All personnel working in the isolation‐free closed‐loop management area (“closed‐loop bubble”) must follow the requirements of closed‐loop management, personal protection, and nucleic acid testing. Inbound individuals whose nucleic acid test results show a *Ct*‐value of 35–40 should be closely observed and retested within 24 h. If the test results show a *Ct*‐value < 35, the individual must be transferred immediately to a designated hospital or a makeshift hospital for treatment; if the test results show a *Ct*‐value ≥ 35, they are generally considered to be previously infected and can be quarantined at home. Quarantined individuals will be assigned a health status code and must not leave their home during the isolation period.

## 4 STRENGTHENING THE OPTIMIZATION AND ADJUSTMENT FROM THE ORGANIZATIONAL LEVEL

To implement optimized and adjusted prevention and control measures, policy interpretation, training and guidance, and delineation of responsibilities need to be emphasized. The goal is to further build consensus across society, avoid and eliminate misinterpretation of optimized and adjusted measures, strengthen leadership and accountability, and achieve the bottom line of preventing widespread epidemics.

### 4.1 Publicity and guidance and policy interpretation

Accurately interpret the adjusted policy, emphasizing adherence to the general strategy of prevention and control of the epidemic in China, and guiding the whole society to fully understand the significance of adhering to the prioritization of human life and the importance of avoiding the importation of foreign infection strains and domestic rebound of COVID‐19. It is necessary to continue to execute a dynamic zero‐COVID policy and reiterate that the further optimization of measures is being undertaken to prevent and control the epidemic more scientifically and precisely. This must not be misinterpreted as a relaxation of the national policy, or misunderstood as giving up the battle against the pandemic. It is necessary to reinforce the understanding that adjustments in policy, which are scientific and necessary, are based on the characteristics of the mutating variants. Trust and support should be gained from the general public and front‐line staff to build a solid foundation for collaborative epidemic response. Public opinion monitoring should be strengthened, and public concerns should be responded to in a timely way.

### 4.2 Supervision and training at all levels

The authorities in charge of each sector should effectively assume responsibility for strengthening the supervision of the adjustment in its field, provide supporting policies, and facilitate the implementation of measures. Local agencies should accurately understand the essence of the policy, refine specific work requirements, and provide training for prevention and control staff at all levels. The purpose of organizing training is to enhance the work capacity of local party committees and governments, joint response mechanisms across various departments, and the front line of prevention and control.

### 4.3 Organizational leadership and delineation of responsibilities

Local party committees and governments at all levels should assume the responsibility of protecting their region. The main person in charge should supervise the division of responsibilities to all parties clearly. According to the real situation, the person in charge should arrange a thorough organizational plan, strengthen training, coordinate supply and human resources, and promote the implementation of the optimization and adjustments of prevention and control measures. We should fully understand the complexity, difficulty, and repetitiveness of the battle against the epidemic, reinforce our responsibility, strengthen our fighting skills, reach to the front line, implement the work of prevention and control of the epidemic, guarantee the production and life of the people, effectively meet the basic needs of the people during the course of the epidemic, guarantee basic livelihood services such as medical care, maintain the normal order of daily life, provide psychological support, and achieve prevention and control of COVID‐19. Bottom‐line and problem‐oriented thinking should be emphasized, on the one hand, to avoid irresponsible attitudes that may result in risk amplification, and, on the other hand, to overcome formalism and bureaucracy and rectify oversimplified or one‐size‐fits‐all approaches and excessive policy steps taken during pandemic control. Optimization and adjustment measures shall be implemented specifically and efficiently to coordinate epidemic response and socioeconomic development and to resonate with the spirit of the 20th Party Congress with practical actions.

The official notice of the policy can be found at: [No. 101 [2022] The Taskforce for Joint Prevention and Control Mechanism for COVID‐19 under the State Council of P.R. China]: http://www.nhc.gov.cn/xcs/zhengcwj/202211/ed9d123bbfe14e738402d846290049ea.shtml.

## AUTHOR CONTRIBUTIONS

**The Taskforce for Joint Prevention and Control Mechanismn for COVID‐19 under the State Council and the National Health Commission of P.R. China**: Conceptualization (lead). **Zongjiu Zhang**: Supervision (lead). **You Wu**: Writing – original draft (lead).

## CONFLICT OF INTEREST

The authors declare no conflict of interest.

## ETHICS STATEMENT

Not applicable.

## INFORMED CONSENT

Not applicable.

## Data Availability

No data involved.
